# 

**Published:** 1915-10-09

**Authors:** 


### CHARITY IN WAR TIME.

The *Hospital Saturday Fund Journal* contains an article on "Charity in Time of War," which shows in an interesting manner how wonderfully successful the appeals for charitable purposes have been in time of war. The millions that have been given as voluntary war gifts do not seem to have diminished the permanent charities. Considering all the circumstances, it is remarkable that the Hospital Saturday Fund should have held its ground so well, and that in the throes of this terrible war its income should be steadily increasing. The income for 1915 exceeds that of the corresponding period of 1914 by some £1,350.

Rates of Subscription: Twelve months, 8/8; Six months, 4/4; Three months, 2/2, post free to any address in the United Kingdom. Abroad, Twelve months, 8/8; Six months, 5/-; Three months, 2/6, post free.—Address The Subscription Dept., "The Hospital," The Hospital Building, 28/29 Southampton Street, Strand, London, W.C.

